# Molecular characterization of a novel Aureusvirus infecting elderberry (*Sambucus nigra* L.)

**DOI:** 10.1371/journal.pone.0200506

**Published:** 2018-08-16

**Authors:** Dana Šafářová, Karolína Vavroušková, Thierry Candresse, Milan Navrátil

**Affiliations:** 1 Department of Cell Biology and Genetics, Faculty of Science, Palacký University, Olomouc, Czech Republic; 2 UMR 1332 Biologie du Fruit et Pathologie, INRA, Univ. Bordeaux, France; McGill University, CANADA

## Abstract

A novel virus infecting elderberry was identified by high-throughput Illumina sequencing of double strand RNAs isolated form elderberry leaves. The complete genome sequence obtained (4512 nucleotides in length) shows an organization typical for aureusviruses, with five open reading frames (ORFs) and the typical ORF1-RT expression by the readthrough of an amber stop codon. The analysis of the RNA-dependent RNA polymerase (RdRp) and coat protein (CP) sequences showed the highest identity (respectively 75.7% and 55%) with the corresponding amino acid sequences of *Pothos latent virus*. These two values, below the species demarcation criteria for the genus, indicate that the detected virus is a new member of genus *Aureusvirus*, family *Tombusviridae*, with the proposed name Elderberry aureusvirus 1 (ElAV1). A survey confirmed the wide distribution of ElAV1 in elderberry in the Czech Republic. Phylogenetic analyses of RdRp and CP sequences showed distinct microevolution of geographically separated isolates, with a tendency for isolates coming from close localities or from the same region to cluster together but heterogeneity of viral populations down to a local scale was also observed. The symptomatology of the new virus is not fully clear, but many infected trees were either asymptomatic or showed mild chlorotic mosaics. More severe symptoms, potentially impacting yields of flowers or berries, were observed in plants with mixed infections of ElAV1 and other elderberry viruses. Further efforts are now needed to determine ElAV1 prevalence outside the Czech Republic and to unravel its epidemiology.

## Introduction

*Aureusvirus* is a genus of single-stranded, positive strand RNA viruses of the family *Tombusviridae*. It was originally established with two members, *Pothos latent virus* (PoLV) as a type species and *Cucumber leaf spot virus* (CLSV) [1]; currently it contains three additional species, *Maize white line mosaic virus* (MWLMV), *Johnsongrass chlorotic stripe mosaic virus* (JCSMV) and *Yam spherical virus* (YSV), as well as the related Sesame necrotic mosaic virus (SNMV), which is not yet formally recognized [2]. Aureusviruses infect various natural host plants but their individual range is generally restricted to a few species, pothos and pigeon pea or lisianthus for PoLV [3–5], yam for YSV [6], sesame for SNMV [7], cucumber, melon or squash for CLSV [1, 8] and various *Poaceae* species, mainly maize (MWLMV, [9, 10]) or johnsongrass (JCSMV, [2, 11]). Aureusviruses are transmitted mechanically and by seeds. Transmission through the soil or by the water circulating in hydroponic systems for PoLV, CLSV and MWLMV, or by the fungus *Olpidium bornovanus* (for CLSV) have also been reported [1, 5, 10–14].

The genomes of aureusviruses are 4.29−4.46 kb long, with an organization typical for *Tombusviridae* members encoding five ORFs, with an uncapped 5´ end and a non-polyadenylated 3´ end. ORF1 encodes a replication-associated protein. Readthrough of an amber stop codon results in the translation of ORF1-RT, which contains an RNA dependent RNA polymerase domain; both proteins are expressed directly and contribute to the replicase complex. The coat protein (CP), encoded by ORF2, and movement protein (MP) encoded by ORF3 are expressed from two distinct subgenomic messenger RNAs (sgRNA1 of 2.0 kDa and sgRNA2 of 0.8 kDa). ORF4, encoding a viral suppressor of silencing, is translated by the leaky scanning of sgRNA2 [1, 2].

Elderberry (*Sambucus nigra* L.) is a deciduous tree native to Europe and North America. Its flowers and berries are used to prepare infusions, syrups and jellies and in traditional medicine. The popularity of this plant has increased in recent years in the pharmaceutical and food industries, due to its antiseptic and antiviral properties as well as to the interest in the colour compounds present in the berries [15, 16]. Several viruses infecting elderberry have been described including carlaviruses (*Betaflexiviridae*) such as *Elderberry carlavirus* (ECV) or *Blueberry scorch virus* (BlScV) [17–19]; nepoviruses (*Secoviridae*) such as *Cherry leaf roll virus* (CLRV), *Arabis mosaic virus* (ArMV), *Tobacco ringspot virus* (TRSV), *Tomato black ring virus* (TBRV) or *Cherry rasp leaf virus* [20–23]; and *Elderberry latent virus* (ELV), *Tobacco necrosis virus* (TNV) or *Tomato bushy stunt virus* (TBSV) in the *Tombusviridae* family [21, 24, 25]. Recently, viruses found to be infecting *Sambucus* plants, and described through next generation sequencing approaches, include elderberry carlaviruses with the proposed name Elderberry virus A–E (*Betaflexiviridae)* [26] and the bromovirus Sambucus virus S (*Bromoviridae*) [27].

## Material and methods

### Biological material

The *Sambucus nigra* L. is a common tree, wildly growing in the Czech Republic. The plant is not protected being classified as B (common) taxon according IUCN and it is not listed in The Red List of the Czech Republic. The all samples were collected in the freely accessible public area and none private land, at any of sampled locations, was visited. None specific permissions were required to perform present study.

Leaf samples were randomly collected from July to September from wild elderberry trees (*Sambucus nigra* L.) during surveys carried out in 2015–2017 in 12 localities of Central Moravia, South Moravia and South Bohemia (Czech Republic). Sampled plants were either asymptomatic or showed various virus-like symptoms, such as leaf chlorosis, chlorotic mosaics and chlorotic spots on leaves, vein clearing, leaf crinkling, rolling and dwarfing, or non-uniform fruit development and ripening. Some trees also showed evidence of early leaf senescence. About 20 g of leaves were collected separately from each plant and subsequently split up into 0.1 and 10 g aliquots that were stored at -80°C until used.

### RNA isolation

Double-stranded RNAs (dsRNAs) were purified from 10 g of leaves of the B15 tree using a double CF11 cellulose batch chromatography procedure [28, 29]. Total RNAs were extracted from 0.1 g of fresh or frozen leaf sample using the NucleoSpin RNA Plant kit (Macherey-Nagel) according the manufacturer recommendations except that leaves were homogenized in a double volume of RA1 buffer using a FastPrep homogenizer. Purified RNAs were eluted in 30 μl of RNAse-free deionized water and kept frozen in -80°C until used.

### NGS and Sanger sequencing

Purified dsRNAs were used for the preparation of a cDNA library using the True Seq Stranded mRNA Library preparation kit (Illumina) and sequenced in a multiplexed run of 100 bp single reads using a HiSeq2500, together with other banks prepared from unrelated plant samples. Both library construction and sequencing were performed by SEQMe s.r.o. (Czech Republic).

For targeted Sanger sequencing of coat protein and RdRp genes or RACE-PCR fragments (at least five clones) all nucleic acids were isolated using the QIAquick Gel Extraction kit (Qiagen). Isolated DNA was sequenced using the BigDye Terminator v3.1 Cycle Sequencing kit (Applied Biosystems) and an ABI PRISM 3730 Genetic analyser.

### RT-PCR virus detection

Total RNAs were used for the detection of the aureusvirus and for the amplification of targeted viral genome parts using RT-PCR. Complementary DNAs were synthetized using random hexamers and the BioScript reverse transcriptase (Bioline). PCR amplification involved the ElAur1F/ElAur1R primer pair targeting the RdRp gene for virus detection (expected PCR fragment of 457 bp; nt positions 716–1172 of the ElAV1-B15 genome, MG967280); the ElAur1-RdRpF/ElAur1-RdRpR primer pair targeting the RdRp domain (1215 bp; nt positions 782–1996); or the ElAur1-CPF/ElAur1-CPR pair targeting the full CP gene (1333 bp, nt positions 2285–3617) (for list of primers see Table 1). These primers were designed based on the NGS data using Primer-BLAST [30]. All PCR amplifications were performed using the MyTaq DNA polymerase (Bioline) and 0.2 μM of each primer. The amplification cycles conditions were: 95°C for 3 min; 35 cycles (95°C for 1 min, 55°C for 1 min, 72°C for 1 min) followed by a final extension step of 72°C for 7 min. PCR amplicons were separated by electrophoresis in 1.5% agarose gel in TAE buffer and visualised with the GelRed nucleic acid stain (Biotium).

**Table 1:** List of primers.

**Name**	**5´ - 3´ sequence**	**Product Size**	**Genome Positions**
***Elderberry aureusvirus 1***			
**ElAur1F**	ATCGACTCGTCATCTTGCCC	457 bp	716–735
**ElAur1R**	GTCATAGCGGGACAGAACCC		1172–1153
**ElAur1-RdRpF**	GTGATGGTGGCTGTTAAGGC	1215 bp	782–801
**ElAur1-RdRpR**	CCTAACCATCACCCACCCTC		1996–1977
**ElAur1-RdRPSeq**	GCTAGTCGYTTYGACCAGCAYTG	-	1537–1559
**ElAur1-CPF**	ATCGCCATTGAGAGGGAGTTG	1333 bp	2285–2305
**ElAur1-CPR**	GTTAAACTTCTTGAGCCACCCA		3617–3596
**5RP-Aur-dir**	CCCAAGCCAACCCGCACCCCCGCC	-	324–301
**5RP-Aur-nt**	TCAGCGTGGCAACAGCCACCACGAGC	-	242–217
**3RP-Aur-dir**	AACACGGACCAAGAGGGTCACACGAACG	-	3505–3532
**3RP-Aur-nt**	ACTCAAGGGATTTAGCACCCAGCCGCG	-	3863–3889
**Name**	**5´ - 3´ sequence**	**Product Size**	**Virus (Reference)**
***Other viruses infecting elderberry***			
**APMV-s**	CGTGAGGAAGTTTAGGTTG	417 bp	ApMV [31]
**APMV-a**	GGCCCCTAAGGGTCATTTC		
**AP1**	AATACCCCGGGTGTTACATCG	419 bp	ArMV [32]
**AP2**	CATTAACTTAAGATCAAGGATTC		
**ArMVnt1**	CCCCAATGATTATTTCCTATGG	187 bp	
**AP2**	CATTAACTTAAGATCAAGGATTC		
**CLRVi1**	GTTAACGAATATCTACTGC	171 bp	CLRV [33]
**CLRVi2**	CAAATATTGCTAAACAACC		
**JQ3D3FF**	GCCAGTTTCTCCAGTGAACC	428 bp	CRLV [34]
**JQ3D3FR**	CAGTTGAACGGATTTAAAC		
**UnicarlaF**	TGYACIGARWSIGAYTRYGARGC	181 bp	ElVA-E [26]
**UnicarlaR**	GCYTCICCISWRWAICKCATDAT		
**1FCPBromo**	ACGAAGGCAGCGAATGGTTA	644 bp	SVS [27]
**1RCPBromo**	CCCGCACTCTAAACCACACT		
**SLRSViF**	TGGCCTTTATTGGTTGGAT	109 bp	SLRSV [33]
**SLRSViR**	ATCTGCCACTGATTCTCAC		
**TBRV-F**	TCGCACTTTGGGGTACAGTC	414 bp	TBRV (this work)
**TBRV-R**	TGGTGGCACACATAATGGCT		

The sequences of the 5´- and 3´-ends of the genome obtained by NGS were verified and completed using the SMARTer RACE 5´/3´ kit (Clontech) and the supplied nested PCR protocol. The 5´-end was obtained using primers 5RPAur-dir followed by 5RPAur-nt, the 3´-end using the 3RPAur-dir and 3RPAur-nt primers, respectively (Table 1). The obtained amplicons were isolated using the QIAquick Gel Extraction kit (Qiagen) and cloned using the pGem-T vector system (Promega).

The potential co-infection of elderberry plants by *Apple mosaic virus* (ApMV), *Arabis mosaic virus* (ArMV), *Cherry leaf roll virus* (CLRV), *Cherry rasp leaf virus* (CRLV), elderberry viruses A-E (ElVA-ElVE), Sambucus virus S (SVS), *Strawberry latent ringspot virus* (SLRSV) and *Tomato black ring virus* (TBRV) was evaluated by RT-PCR reactions performed on cDNAs obtained as described above. The following specific primer pairs and original protocols were used: APMV-s and APMV-a for ApMV [31], AP1 and AP2 followed by ArMVnt1 and AP2 in semi-nested PCR for ArMV [32], CLRVi1 for CLRVi2 for CLRV [33], JQ3D3FF and JQ3D3FR for CRLV [34], UnicarlaF and UnicarlaR for elderberry carlaviruses [26], 1FCPBromo and 1RCPBromo for SVS [27], SLRSViF and SLRSViR for SLRSV [33] and TBRV-F and TBRV-R for TBRV (for the summary see Table 1).

### Bioinformatic analysis of sequence data

*De novo* assembly of high quality reads was performed with the CLC Genomics Workbench 9, using default parameters and a minimum contig length of 200 bp. The obtained contigs were annotated by BLASTN and BLASTX analyses [35] against the GenBank nt and nr databases [36]. Sanger sequencing reads were assembled using the Seqman Pro Assembler, Lasergene v. 12.3.1.4 (DNASTAR, Inc.).

Multiple alignments, pairwise genetic distance computations, model selections, all phylogenetic analyses and tree visualisations were performed using Mega v. 7.0.14 [37]. The obtained full genomic sequence and/or partial sequences were aligned with all available GenBank aureusvirus sequences and with reference *Tombusviridae* sequences using the ClustalW algorithm [38]. Phylogenetic analyses were performed using the neighbor-joining method [39, 40] and the Tamura 3-parameter model [41] for nucleotide (nt) sequences or the JTT model [42] for amino acid (aa) sequences with 500 bootstrap replications. The final phylogenetic trees were visualised using Tree Explorer [37]. Conserved protein viral domains were identified using NCBI BLAST-CDD [43, 44]. The obtained sequences have been deposited in NCBI GenBank [36] under accession numbers MG967280-MG967323.

## Results

**Fig 1:**
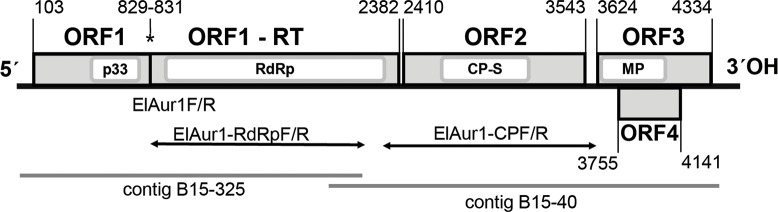
Schematic genome organization of Elderberry aureusvirus 1 depicting also the position on the genome of the NGS de novo contigs and of the PCR products used in the present study. Conserved amino acid domains are indicated by white boxes: p33: tombusviridae p33 domain, RdRp: RNA dependent RNA polymerase domain, CP-S: coat protein S domain, MP: movement protein domain. The readthrough amber stop codon is indicated by an asterisk and the nucleotide position on the genome of the various ORFs indicated above the diagram. Arrows mark the position of specific RT-PCR amplicons used for isolates genetic diversity characterisation.

During the survey for the viruses infecting elderberry trees in the Czech Republic, a tree (marked as B15) showing virus-like symptoms such as leaf chlorosis, leaf dwarfing and reduced, non-uniform fruit development, was selected for detailed NGS analysis. Highly purified double-stranded RNAs were sequenced using the Illumina HiSeq2500 platform. In total, 27,747,697 reads (average length 100 nt) were obtained and were assembled in 4638 contigs, two of which showed high identity values with aureusvirus sequences available in GenBank database. Contig B15-325, 2137 nt in length (1346 reads, average coverage 60x) showed 78.2% identity with the RNA-dependent RNA polymerase gene of *Pothos latent virus* (PoLV, isolate lisianthus, AB602348); contig B15-40, 2436 nt in length (1801 reads, 73x coverage) showed 83.2% identity with the partial coat protein sequence of PoLV-pigeonpea (AJ243370). The scaffolding of the overlapping ends these contigs (Fig 1), together with 5´-RACE and 3´-RACE PCR amplifications allowed us to obtain the complete genome sequence of the virus infecting the original B15 elderberry tree.

The B15 isolate genome is 4512 nt long (3144 reads, average coverage 69x) and comprises five ORFs with the typical organisation of aureusviruses in the *Tombusviridae* family (Fig 1). The first open reading frame is 729 nt long (genome positions 103–831) and encodes a replication-associated protein of 242 aa (26.5 kDa) with a typical tombusvirus p33 protein domain (nt positions 463–765; aa ORF positions 121–221). The ORF1-RT (positions 103–2382) is 2280 nt long and readthrough of the stop amber codon (position 829–831) allows the expression of the RNA-dependent RNA polymerase of 758 aa (85.3 kDa). ORF1-RT harbours a typical RdRp domain (nt positions 928–2295; aa positions 276–731). ORF2 is 1134 nt long (genome positions 2410–3543) and encodes the coat protein of 377 aa (39.6 kDa) with its conserved viral CP S domain (nt positions 2650–3195, aa ORF positions 81–262). ORF3 is 711 nt long (genome positions 3624–4334) and encodes a movement protein of 236 aa (26 kDa) with a conserved core protein P21/22 domain (nt positions 3639–4058; aa ORF positions 6–145). ORF4 is 387 nt long (genome positions 3755–4141) and encodes an RNA silencing suppressor of 128 aa (14 kDa).

A BLAST analysis of the nucleotide sequence (nt) of the RdRp-encoding region showed highest identity (70.6%) with the aureusviruses *Pothos latent virus* isolate pigeonpea (AJ243370) and *Yam spherical virus* (NC_022895). Comparisons of the deduced amino acid sequence showed respectively 75.7% and 75.4% identity with the RdRps of PoLV-pigeonpea and YSV. The highest aa identity (78.7%) was found with the partial RdRp of Sesame necrotic mosaic virus (DQ367845). A similar analysis of CP gene sequences confirmed the genetic differentiation of the B15 virus, with highest nucleotide identity levels of respectively 60.3% and 58.4% (55% and 54.2% aa-identity) with PoLV isolates lisianthus and pigeonpea (AB602348, AJ243370) (for detailed analyses see S1 Table).

**Fig 2:**
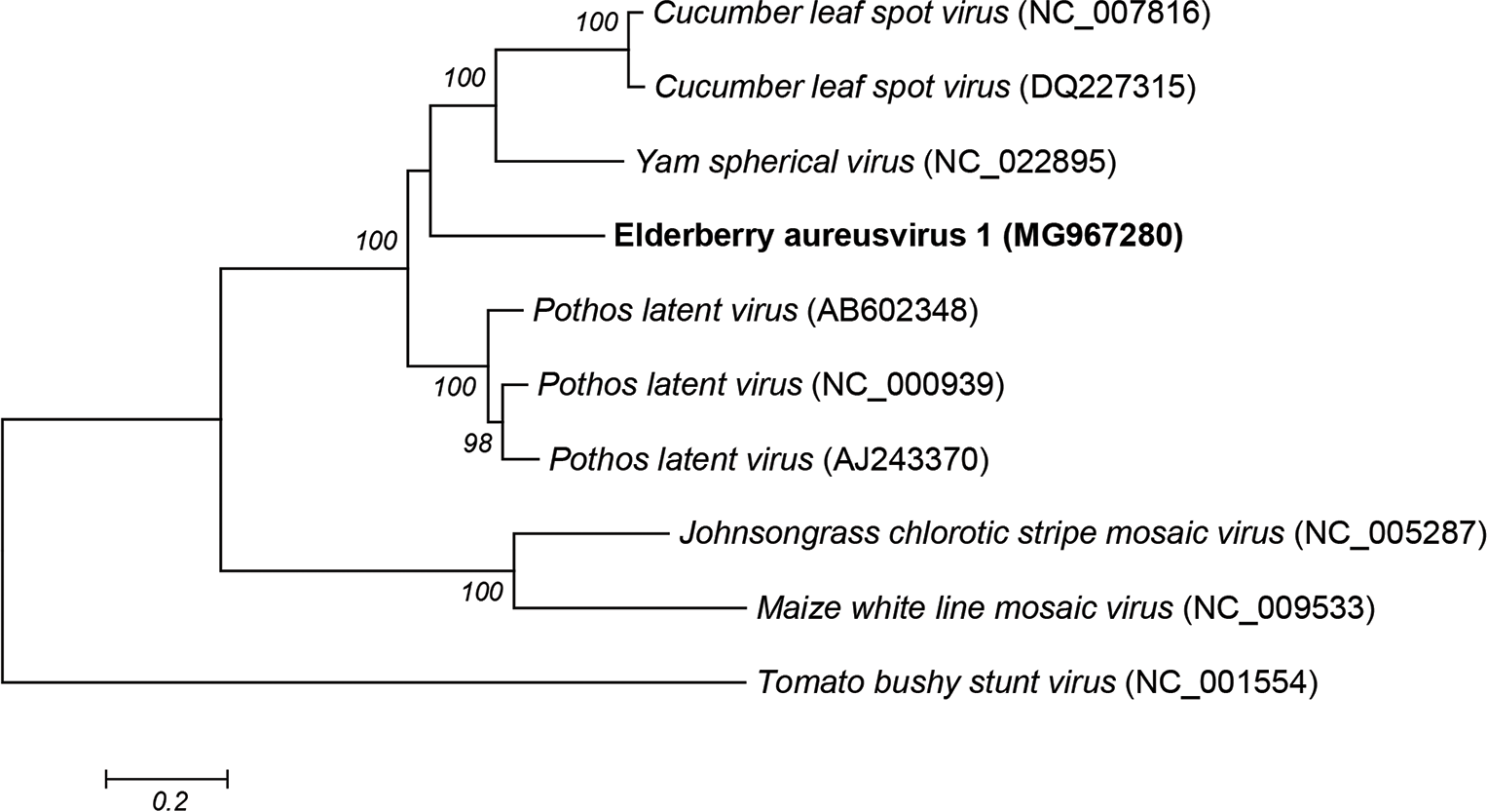
Neighbor-joining phylogenetic tree reconstructed using the complete genome sequences of Aureusvirus genus members. Virus name and GenBank accession number in parenthesis are indicated. Tomato bushy stunt virus was used as an outgroup to root the tree. Bootstrap values ≥70% (500 replicates) are shown. Elderberry aureusvirus 1 is marked in bold. The scale bar represents 20% nucleotide sequence divergence.

All observed aa-identities are therefore below the species demarcation criteria in the genus *Aureusvirus*, i.e. less than 60% aa sequence identity for the CP and less than 80% aa sequence identity for the polymerase. This clearly indicates that isolate B15 represents a new aureusvirus species, tentatively named Elderberry aureusvirus 1 (ElAV1). Comparison of the available complete genomic sequences of aureusviruses and reference species within the family *Tombusviridae* and their phylogenetic analysis confirmed the position of the new virus in the *Aureusvirus* genus, with the B15 isolate forming a separate branch in the close relation to PoLV and YSV (Fig 2).

### ElAV1 detection, distribution and symptomatology

To obtain information on the prevalence of the novel aureusvirus, a random screening of elderberry trees growing at twelve localities of three different Czech regions, i.e. Central Moravia, South Moravia and South Bohemia was performed during 2015–2017. RT-PCR detection primers ElAur1F and ElAur1R were designed based on the full genome sequence of the B15 isolate. They target a region covering part of the putative p33 and RdRp domains (genome positions 716–1172 of the ElAV1-B15 isolate, MG967280). The expected product of 457 bp (S1 Fig) was amplified from 31 of the 95 tested trees, demonstrating the presence of the virus in all three sampled regions at an overall prevalence of 32.6%.

**Fig 3:**
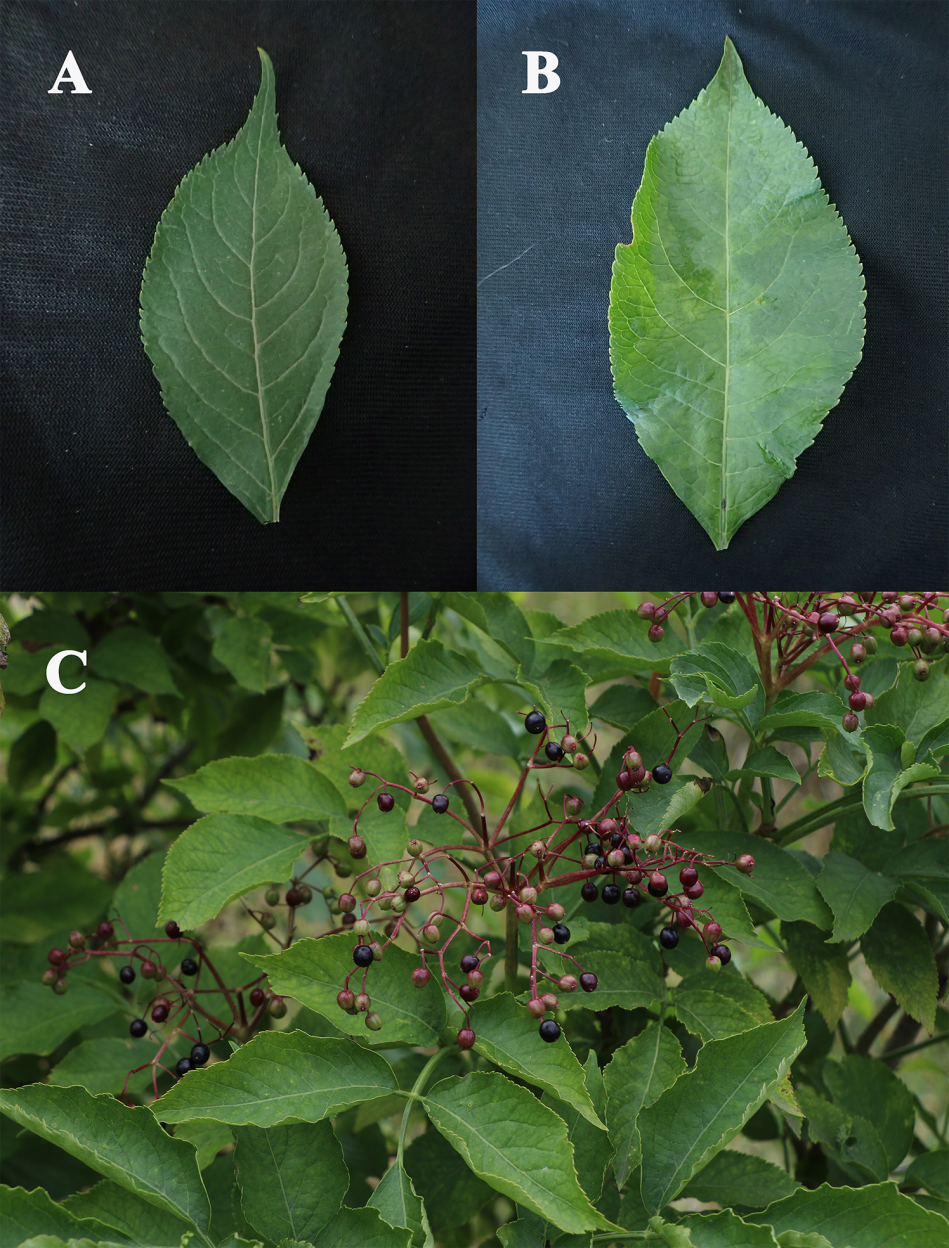
Symptoms observed on elderberry aureusvirus 1 infected elderberry trees. (A) non-symptomatic leaf, (B) mild chlorotic mosaics on leaf, (C) non-homogeneous fruit development and mild chlorosis on leaves.

The trees infected by Elderberry aureus virus 1 were either asymptomatic or showed mild symptoms of virus infection, mainly mild chlorosis or diffuse chlorotic mosaics on the leaves. In a few cases a non-uniform flowering or fruit ripening was also observed (Fig 3). In some cases, more severe symptoms (chlorosis, chlorotic ring spots) were also observed, but these were always associated with mixed infections involving other viruses such as ElVA, ElVB and/or CLRV. More rarely, TBRV and ArMV were also detected in mixed infection with Elderberry aureusvirus 1 (for detailed summary see Table 2).

**Table 2:** Virus indexing of ElAV1 infected plants and observed symptoms.

Isolate	Detected virus								Observed symptoms
	ElAV1	ApMV	ArMV	CLRV	CRLV	ElV	SVS	TBRV	
**B1**	**+**	-	-	**+**	-	-	-	-	mild chlorosis, leaf rolling
**B5**	**+**	-	-	**+**	-	**+**	-	-	chlorosis
**B6**	**+**	-	-	-	-	-	-	-	none symptoms
**B7**	**+**	-	-	-	-	**+**	-	-	diffuse chlorotic mosaics
**B10**	**+**	-	-	-	-	**+**	-	-	none symptoms
**B11**	**+**	-	-	-	-	**+**	-	-	chlorotic ring spots
**B12**	**+**	-	-	-	-	**+**	-	-	chlorosis
**B13**	**+**	-	-	-	-	-	-	-	none symptoms
**B15**	**+**	-	-	**+**	-	**+**	+	-	chlorosis, leaf dwarfing, non-uniform fruit development
**B17**	**+**	-	-	-	-	-	-	-	none symptoms
**B18**	**+**	-	-	-	-	-	-	-	mild chlorotic mosaics
**B19**	**+**	-	-	-	-	-	-	-	none symptoms
**B22**	**+**	-	**+**	**+**	-	**+**	-	**+**	chlorotic ring spots
**B25**	**+**	-	-	-	-	**+**	-	-	chlorotic ring spots
**B29**	**+**	-	**+**	-	-	**+**	-	-	chlorotic ring spots
**B30**	**+**	-	**+**	**+**	-	-	-	-	diffuse chlorotic mosaics
**B32**	**+**	-	-	**+**	-	-	-	-	chlorotic ring spots
**B71**	**+**	-	-	-	-	**+**	-	-	diffuse chlorotic mosaics
**B73**	**+**	-	-	-	-	**+**	-	-	none symptoms
**B74**	**+**	-	-	-	-	**+**	-	-	leaf crinkling
**B75**	**+**	-	-	-	-	+	-	-	chlorotic mosaics
**B76**	**+**	-	-	-	-	**+**	-	-	none symptoms
**B79**	**+**	-	-	-	-	**+**	-	-	none symptoms
**B80**	**+**	-	-	-	-	+	-	-	chlorotic mosaics
**B81**	**+**	-	-	-	-	-	-	-	none symptoms
**B83**	**+**	-	-	-	-	**+**	-	-	diffuse chlorotic mosaics
**B153**	**+**	-	-	**+**	-	+	-	-	chlorosis, leaf dwarfing
**B158**	**+**	-	-		-	+	-	-	chlorosis
**B159**	**+**	-	-	**+**	-	+	-	-	chlorosis, leaf dwarfing
**B259**	**+**	-	-	-	-	+	-	-	chlorosis, leaf dwarfing, non-uniform fruit development
**B260**	**+**	-	-	-	-	+	-	-	chlorosis, leaf dwarfing

ElAV1 –Elderberry aureusvirus 1, ApMV–*Apple mosaic virus*, ArMV–*Arabis mosaic virus*, CLRV–*Cherry leaf roll virus*, CRLV–*Cherry rasp leaf virus*, ElV–Elderberry viruses A-E, SVS–Sambucus virus S, TBRV–*Tomato black ring virus*; (+) positive plant, (-) negative plant.

### Genetic variability of the elderberry aureusvirus 1

**Fig 4:**
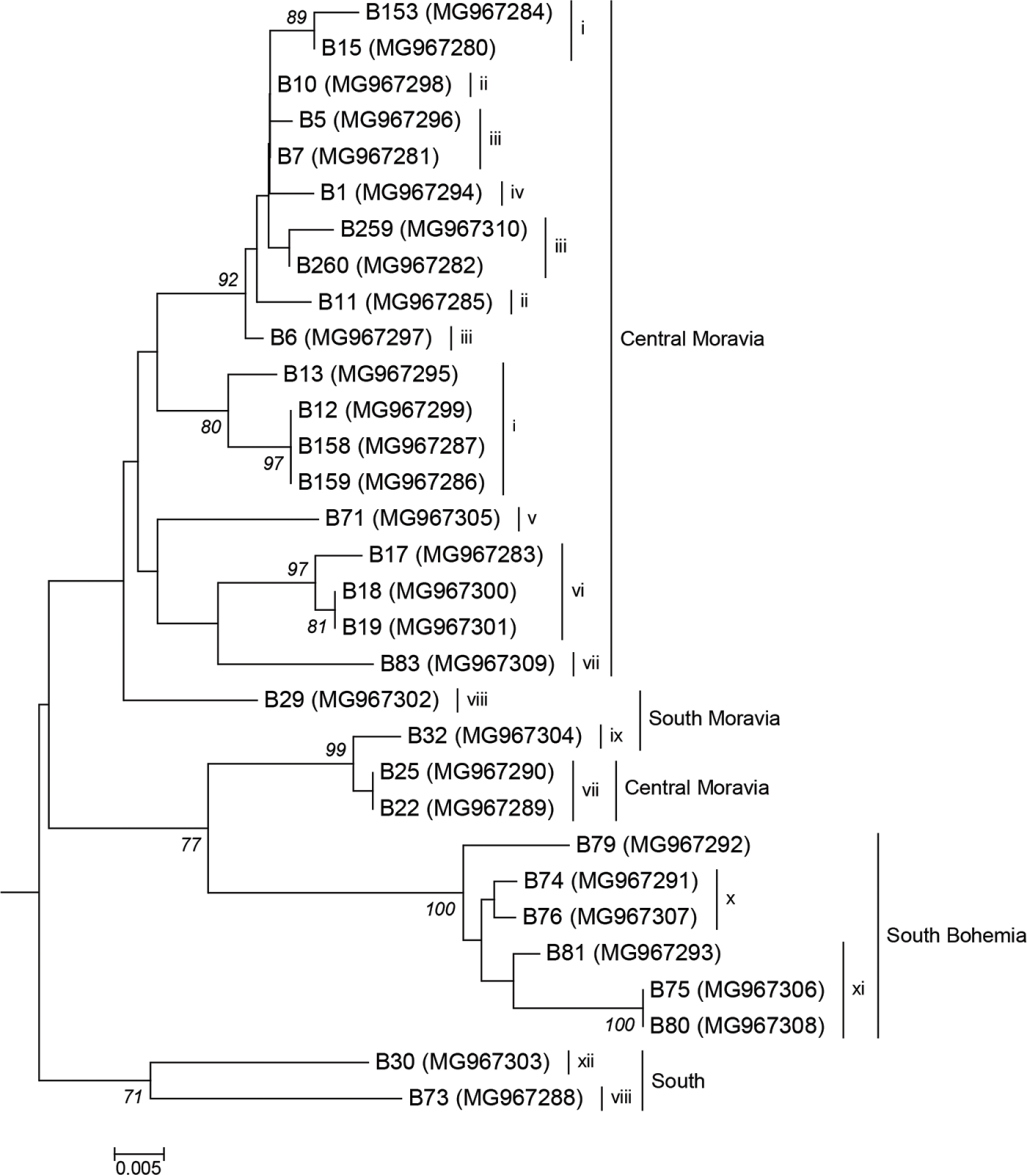
Neighbor-joining phylogenetic tree based on the ElAur1F/R RdRp nucleotide sequences of elderberry aureusvirus 1 isolates. PoLV was used as an outgroup to root the tree. Bootstrap values ≥70% obtained from 500 replicates are shown. The scale bar represents 0.5% nt sequence divergence. The locality of origin of the isolates is indicated as follows: i—Slatinky, ii–Hněvotín, iii–Hejčín, iv–Olomouc, v–Slatinice, vi–Rostěnice, vii–Náměšť na Hané, viii–Čejkovice, ix–Milotice, x–Střelské Hoštice, xi–Veřechov, xii–Kloboučky.

Sequence analysis of the ElAur1F/R amplicons confirmed the specificity and robustness of the detection assay, as no non-specific PCR products were identified. All isolates, including the ElAV1-B15 reference isolate, shared 91.2–99.8% nt identity (93.3–100% amino acid identity of the encoded protein). A phylogenetic analysis of these nucleotide sequences confirmed the significant variability of the virus and showed the existence of several well supported clusters but also of isolated sequences at the end of long branches (Fig 4). The isolates from South Bohemia formed a separate cluster while those from South and Central Moravia separated in several groups, although the majority of the isolates from Central Moravia formed a large cluster. In several cases, isolates sampled from the same locality were separated in different clusters, indicating the heterogeneity of viral populations down to a local scale. This was for example the case of the isolates collected in Slatinky, the locality in which the ElAV1-B15 isolate was detected by NGS, and for which the existence of two distinct subclusters was observed (Fig 4).

To further analyse the genetic variability of the new aureusvirus, the sequence of two genomic regions, the RdRp and the CP were analysed. For the first one, a sequence covering almost the full RdRp of ORF1-RT (1258 bp) was assembled from two overlapping PCR fragments amplified using the ElAur1F/ElAur1R and ElAur1-RdRpF/R primer pairs. For the second one, the complete sequence of the coat protein gene (1134 bp) was obtained from the ElAur1-CPF/R amplicon. Despite the fact, that the primers used were designed based on the B15 isolate sequence only, both sequences were successfully obtained for 14 isolates coming from all Czech regions. ElAV1 isolates showed higher variability in the RdRp domain, with 90.6–99.8% nucleotide identity (95.5–100% aa identity) to be compared to 95.5–100% nucleotide identity (96.8–100% aa identity) for the CP gene.

Phylogenetic analyses confirmed the diversity of the virus and the tendency for isolates from the same region to cluster together as one or two main groups (S2A and S2B Fig). In particular, the ORF1 analysis supported the previous discrimination of the South Bohemian isolates (B74, B76, B79, B80 and B81) and of two isolates from Central Moravia (B22 and B25) into two clusters significantly different from other isolates. The same pattern was also observed when analysing the CP gene.

## Discussion

Elderberry is a widely growing shrub or tree, popular in the central Europe and in other areas due to the efficacious or useful compounds present in its fruits and flowers. Not surprisingly, in agreement with its use in traditional medicine, elderberry was a center of interest for virologists in the former Czechoslovakia, and in other countries too, resulting in the description of various diseases and virus-like infections based only on the observation of symptomatology and/or supported by transmission experiments to differential or indicator hosts and by electron microscopy [23, 25, 45, 46]. Despite the availability of detailed descriptions including photodocumentation, the viruses responsible for these syndromes have not been deposited or maintained in collection until now, severely complicating their identification.

Next generation sequencing is proving a very powerful tool for the description of new viruses and for solving etiological issues. Until recently various elderberry carlaviruses described based on viral particle morphology and on detection assays using polyclonal antibodies could be found in the virological literature, named as Elderberry virus A, Elderberry carlavirus, and finally re-named as Elderberry symptomless virus [17, 21, 24, 46, 47]. Given the uncertainties about the precise identity of the agent(s) thus named, and due to the lack of authentic material [47], these virus taxons and names have been abolished by the International Committee on Taxonomy of Viruses. Interestingly, the newly recognized elderberry carlavirus species, Elderberry virus A-E (ElVA-E), that apparently show similar symptoms as the abolished ones, were proposed on the basis of NGS data, with the authors suggesting that the previously reported elderberry carlaviruses may have represented unique or mixed infections of various ElVA-E isolates [26]. Recently, NGS was also successfully used for the detection and characterization of a new bromovirus infecting European elderberry, Sambucus virus S [27].

Elderberry aureusvirus 1, a novel aureusvirus in the family *Tombusviridae*, detected and characterized here, shows that the elderberry virome is wider than was expected. The family *Tombusviridae* comprises 16 genera, and three viral species belonging to different *Tombusviridae* genera, ELV (*Pelarspovirus*), TNV (*Necrovirus*) and ToBSV (*Tombusvirus*), have been reported to unfrequently infect elderberry [21, 24, 25]. To our best knowledge there are no reports of observations of viral particles or of other elements that might indicate the presence of the other viruses belonging to this family in elderberry plants. Thus, the detected new virus not only widens the number of viruses known to infect elderberry but is also widening the range of *Tombusviridae* species, representing the sixth member of *Aureusvirus* genus.

The presence of virus in various randomly sampled trees detected using RT-PCR and confirmed by subsequent sequencing shows that ElAV1 is widely distributed, at least in the Czech Republic. The detected genetic diversity suggests that the virus has been present in these regions for a long time. Aureusviruses generally have a limited natural host range [2] and our observations tend to confirm this trend, since ElAV1 was only detected in elderberry plants but not in the other plants we analysed (such as maple, wild rose, blackberry or raspberry, cornel, sorbus, nettle, bindweed, bedstraw and fleabane) growing in the surroundings or in the green cover under infected trees.

The symptomatology of the new aureusvirus in infected elderberry trees is not fully clear. Despite the mild chlorosis and mild or diffuse mosaics observed on the leaves of many infected trees, these symptoms cannot be unambiguously connected with the virus. Elderberry trees prefer humid soils rich in nitrogen and phosphorus, but they tolerate a wide range of soil types and often grow in villages and towns at ruderal sites, around roads or streams [16, 48]. It cannot be excluded that the observed mild chlorosis or even non-uniform fruit development could result from abiotic physiological stress induced by nutrient or water deficiencies, or by soil pH. Based on the observation that many infected trees show mild or no symptoms and that trees with severe symptoms are systematically co-infected with other viruses, it is likely that ElAV1 infection has limited detrimental effects on elderberry plants, possibly suggesting a long evolutional co-adaptation of the virus and its elderberry hosts. However, the existence of very sensitive elderberry genotypes cannot be excluded. In addition, ElAV1 could significantly impact to the health status of wild or cultivated elderberry plants in the case of mixed infections with other viral agents. A correlation between severe symptoms of virus infection and mixed infections of carlaviruses, nepoviruses and/or secoviruses has already been reported [17, 25, 49]. In this respect, the results of our survey repeatedly showed that elderberry infection by ElAV1 alone or by elderberry carlaviruses (ElV-A and B) alone was associated with no or with weak symptoms but that the trees co-infected by these agents showed more severe chlorotic mosaics and leaf chlorosis as well as non-regular fruit development.

The epidemiology of ElAV1 still remains unclear. Efforts at experimental transmission of ElAV1 to *Nicotiana benthamiana* and *Chenopodium quinoa* by mechanical inoculation have been unsuccessful so far, although the closely related *Pothos latent virus* is reported to be mechanically transmissible to these herbaceous hosts [1, 5, 12]. The evaluation of other transmission mechanisms typical for aureusviruses, by seeds or through the soil and/or soil-borne vector(s) is under progress.

## Supporting information

S1 Table Elderberry aureusvirus 1 nucleotide and amino acid sequence identities with aureusviruses and other close related *Tombusviridae* species. (XLSX)

S1 Fig Detection of Elderberry aureusvirus 1 by ElAur1F/R RT-PCR in various elderberry trees. Lanes: L, GeneRuler 100 bp DNA ladder (Thermo Scientific); 1–15, elderberry samples.
(PDF)

S2 Fig **Phylogenetic tree of Elderberry aureusvirus 1 isolates reconstructed using neighbor-joining method based on the RdRp (A) and complete coat (B) proteins.** PoLV used as an outgroup to root the tree. Bootstrap values ≥70% obtained from 500 replicates are shown. The scale bars represent 0.5% sequence divergence. Isolates are marked by name and GenBank Accession number.
(PDF)
