# Supplementary material for: Molecular characterization of a novel Aureusvirus infecting elderberry (Sambucus nigra L.)
Source: PLoS One. 2018 Aug 16;13(8):e0200506. doi: 10.1371/journal.pone.0200506 (PMC6095521; doi:10.1371/journal.pone.0200506)

S1 Fig. Detection of Elderberry aureusvirus 1 by ElAur1F/R RT-PCR in various elderberry trees. Lanes: L, GeneRuler 100 bp DNA ladder (Thermo Scientific); 1-15, elderberry samples.

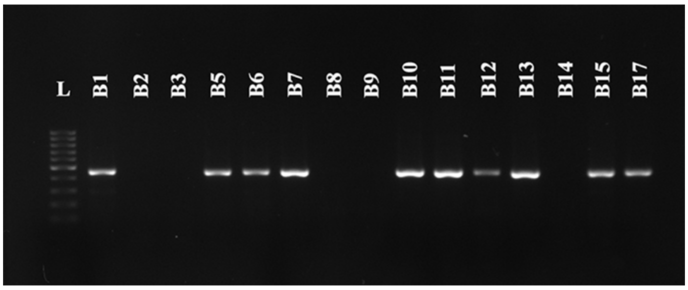

Supplement: S1 Fig — Lanes: L, GeneRuler 100 bp DNA ladder (Thermo Scientific); 1–15, elderberry samples. (PDF) [file pone.0200506.s002.pdf]
