# Supplementary material for: Molecular characterization of a novel Aureusvirus infecting elderberry (Sambucus nigra L.)
Source: PLoS One. 2018 Aug 16;13(8):e0200506. doi: 10.1371/journal.pone.0200506 (PMC6095521; doi:10.1371/journal.pone.0200506)

S2 Fig. Phylogenetic tree of Elderberry aureusvirus 1 isolates reconstructed using neighbor-joining method based on the RdRp (A) and complete coat (B) proteins. PoLV used as an outgroup to root the tree. Bootstrap values ≥70% obtained from 500 replicates are shown. The scale bars represent 0.5% sequence divergence. Isolates are marked by name and GenBank Accession number.

A

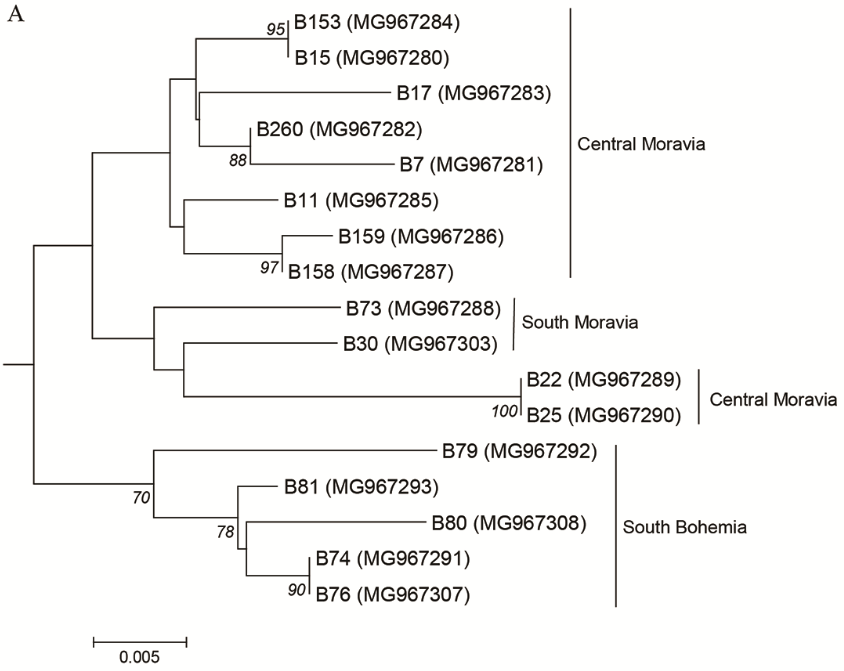

B

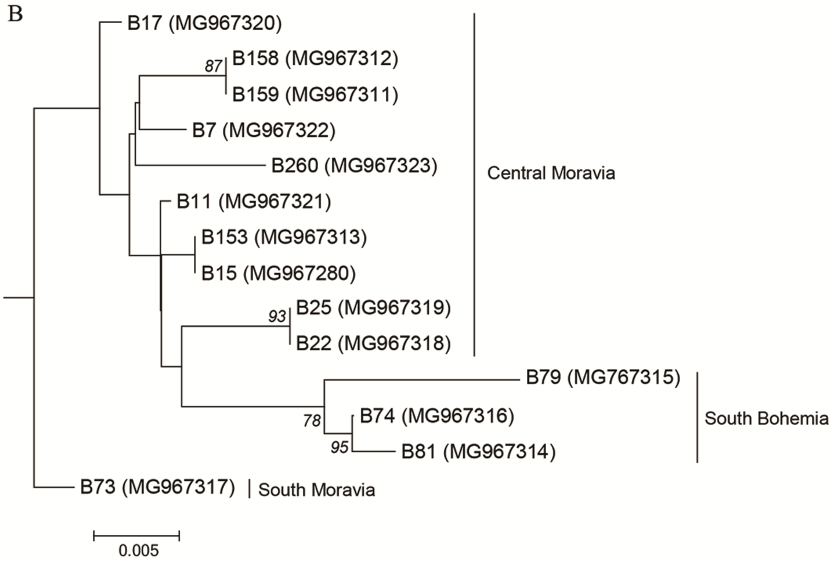

Supplement: S2 Fig — Phylogenetic tree of Elderberry aureusvirus 1 isolates reconstructed using neighbor-joining method based on the RdRp (A) and complete coat (B) proteins. PoLV used as an outgroup to root the tree. Bootstrap values ≥70% obtained from 500 replicates are shown. The scale bars represent 0.5% sequence divergence. Isolates are marked by name and GenBank Accession number. (PDF) [file pone.0200506.s003.pdf]
